# The Contribution of Proprioceptive Information to Postural Control in Elderly and Patients with Parkinson’s Disease with a History of Falls

**DOI:** 10.3389/fnhum.2014.00939

**Published:** 2014-11-24

**Authors:** Esther M. J. Bekkers, Kim Dockx, Elke Heremans, Sarah Vercruysse, Sabine M. P. Verschueren, Anat Mirelman, Alice Nieuwboer

**Affiliations:** ^1^Neuromotor Rehabilitation Research Group, Department of Rehabilitation Sciences, KU Leuven, Leuven, Belgium; ^2^Musculoskeletal Rehabilitation Research Group, Department of Rehabilitation Sciences, KU Leuven, Leuven, Belgium; ^3^Movement Disorders Unit, Department of Neurology, Tel-Aviv Sourasky Medical Center, Tel-Aviv, Israel

**Keywords:** Parkinson’s disease, falls, proprioception, aging, postural control

## Abstract

Proprioceptive deficits negatively affect postural control but their precise contribution to postural instability in Parkinson’s disease (PD) is unclear. We investigated if proprioceptive manipulations differentially affect balance, measured by force plates, during quiet standing in 13 PD patients and 13 age-matched controls with a history of falls. Perceived limits of stability (LoS) were derived from the differences between maximal center of pressure (CoP) displacement in anterior–posterior (AP) and medio-lateral (ML) direction during a maximal leaning task. Task conditions comprised standing with eyes open (EO) and eyes closed (EC): (1) on a stable surface; (2) an unstable surface; and (3) with Achilles tendon vibration. CoP displacements were calculated as a percentage of their respective LoS. Perceived LoS did not differ between groups. PD patients showed greater ML CoP displacement than elderly fallers (EF) across all conditions (*p* = 0.043) and tended to have higher postural sway in relation to the LoS (*p* = 0.050). Both groups performed worse on an unstable surface and during tendon vibration compared to standing on a stable surface with EO and even more so with EC. Both PD and EF had more AP sway in all conditions with EC compared to EO (*p* < 0.001) and showed increased CoP displacements when relying on proprioception only compared to standing with normal sensory input. This implies a similar role of the proprioceptive system in postural control in fallers with and without PD. PD fallers showed higher ML sway after sensory manipulations, as a result of which these values approached their perceived LoS more closely than in EF. We conclude that despite a similar fall history, PD patients showed more ML instability than EF, irrespective of sensory manipulation, but had a similar reliance on ankle proprioception. Hence, we recommend that rehabilitation and fall prevention for PD should focus on motor rather than on sensory aspects.

## Introduction

Falls are a major problem in the elderly population. Approximately 30% of community-dwelling elderly over 65 years of age experience at least one fall per year and this amount increases up to 50% by the age of 80 (Tinetti and Williams, 1997). In patients with Parkinson’s disease (PD), the occurrence of falls is even higher, with 61% (range 35–90%) reporting at least one fall per year (Allen et al., 2013). As falls may lead to injuries (Bloem et al., 2001), fear of falling (Adkin et al., 2003), restriction of daily activities (Bloem et al., 2001), and mortality (Kannus et al., 1999), their impact poses large economic and societal burdens to health care systems worldwide (Muir et al., 2013).

Maintaining balance requires a complex interplay between sensory and motor systems (Horak et al., 2009; Zwergal et al., 2012; Muir et al., 2013). To maintain a stable upright stance, information processed through the somatosensory (70%), visual (10%), and vestibular (20%) system needs to be integrated (Horak, 2006). With increasing age, the integrity of these systems declines (Horak, 2006; Muir et al., 2013), resulting in sensory impairments and gait and balance disturbances (Dozza et al., 2005; Cho et al., 2012). Quiet standing predominantly depends on somatosensory processing, with proprioception, or the sense of body movement and spatial orientation, as a vital component. Accordingly, age-related proprioceptive deficits have been associated with loss of static postural stability and falling (Lord et al., 1991; Goble et al., 2009, 2011). Moreover, disrupting proprioception during quiet standing on a movable surface was shown to increase body sway in the elderly and subjects with sensory polyneuropathy (Horak et al., 2002; Speers et al., 2002; Goble et al., 2011). Postural sway is defined as the continuous movement of the body’s center of mass and can be measured by the use of force plates through detecting fluctuations of the center of pressure (CoP) (Schoneburg et al., 2013) under static or dynamic conditions (Nonnekes et al., 2013). The proprioceptive dependence during balance control can be investigated by changing the stability of the surface or adding ankle tendon vibration during stance. Standing on a foam surface reduces the proprioceptive input from the ankle and the cutaneous inputs from the soles (Lord et al., 1991), whereas, tendon vibration stimulates Ia afferent nerves and modifies proprioceptive information from the vibrated muscle, evoking an illusion of movement (Vaugoyeau et al., 2011). At the Achilles tendon level, the vibrated muscle is perceived to be longer than it actually is, causing an illusory forward body displacement (Brumagne et al., 2000; Vaugoyeau et al., 2011). In order to compensate for this, the body will be inclined backwards, leading to a backward CoP displacement (Vaugoyeau et al., 2011). Older adults and patients with low back pain have altered proprioceptive sensitivity to vibration in the lower back and ankles compared to healthy adults (Brumagne et al., 2000, 2004). Both groups showed a larger response to ankle vibration indicating an increased reliance on ankle proprioception and use of an ankle postural strategy (Brumagne et al., 2004). This ankle strategy can be described as simplifying the body as an inverted pendulum pivoting at the ankles, an effective strategy during static conditions (Boonstra et al., 2013) but inadequate to prevent postural instability and falls during dynamic conditions with higher postural demands. In these latter conditions, switching to an increased use of arm, hip, or knee function is needed to achieve a stable posture. In this respect, it has been suggested that PD patients experience difficulties switching between postural strategies and that this inflexibility may reflect an important role of the basal ganglia, which is disturbed in PD (Schoneburg et al., 2013). Additionally, it has been shown that Achilles tendon vibration disturbed postural control more in sedentary elderly than in active elderly (Maitre et al., 2014).

The use of tendon vibration during functional magnetic resonance imaging (fMRI) has led to a better understanding of the neural basis of central proprioceptive feedback processing and how it is affected by aging (Goble et al., 2012). Many basal ganglia neurons have proprioceptive receptive fields, which indicates that these nuclei play an important role in the sensorimotor integration, i.e., the mechanisms by which sensory information is processed to guide motor planning and execution, and have shown reduced activity in older compared to younger subjects (Konczak et al., 2009; Suetterlin and Sayer, 2013). Goble et al. (2011) confirmed a link between proprioception-related neural activity in basal ganglia structures and balance performance using ankle tendon vibration, whereby increased activation of the pallidum and putamen was associated with better balance performance, although no age differences were found.

Basal ganglia dysfunction is the hallmark of the pathophysiology of PD. Consequently, proprioceptive deficits may contribute to the impaired postural control found in this group. It has been suggested by previous studies that PD patients show smaller limits of stability (LoS) (Horak et al., 2005; Mancini et al., 2008; Menant et al., 2011) and higher medio-lateral (ML) sway (Melzer et al., 2004; Mancini et al., 2012) than healthy elderly. In addition, the duration of the disease and the presence of freezing of gait (FOG) were identified as factors that may impact upon postural instability in PD (Menant et al., 2011). Postural instability, history of falls and cognitive impairment were identified as important risk factors of future falls in PD (Allen et al., 2013). Moreover, fallers with PD showed poorer peripheral sensation (Kerr et al., 2010), reduced lower limb strength, and impaired visual contrast sensitivity compared to PD non-fallers (Latt et al., 2009). Proprioceptive dysfunction in PD was shown to not only affect postural instability by impairing adaption to a changing base-of-support (Chong et al., 2000), but also by reducing the accuracy of compensatory stepping, the coupling between postural adjustments and voluntary movement, the perception of trunk and surface orientation and postural sway in stance [for review see Konczak et al. (2009)].

The abovementioned evidence suggests that proprioceptive deficits may affect postural stability in patients with PD and elderly differently. However, the difference between patients with PD and elderly fallers (EF) has not been assessed. Therefore, we compared PD fallers with EF rather than healthy elderly to understand the specific contribution of the basal ganglia to sensory-system-induced postural instability. Only few studies on postural control have investigated proprioceptive manipulations using tendon vibration with and without visual feedback in PD, and results are inconclusive (Smiley-Oyen et al., 2002; Vaugoyeau et al., 2011). The present study aims to clarify differences in the reliance upon proprioceptive information in postural control in fallers with and without PD by use of tendon vibration, and stable and unstable surfaces. Based on the above-cited literature, we expected a greater impact of proprioceptive perturbations meaning more use of an ankle-steered postural control, leading to greater postural sway in the PD group than in the EF cohort and this more so in conditions without visual feedback.

## Materials and Methods

### Participants

Twenty-six participants were investigated, including 13 patients with PD (9 male and 4 female) with a history of falls and 13 age-matched healthy EF (4 male and 9 female). Participants were included if they had Mini-Mental State Examination (MMSE) scores ≥24, were free of other neurological diseases than PD and of other comorbidity affecting postural instability, were able to stand upright independently and experienced at least one fall in the previous 6 months. A fall was defined as “an unexpected event in which a person comes to rest on the ground, floor or lower level” (Lamb et al., 2005). All PD patients were recruited from the Movement Disorders Clinic of the University of Leuven and all participants gave informed consent according to the Declaration of Helsinki. The study was approved by the Medical Ethics Committee of the University Hospitals Leuven.

### Procedure

#### Clinical assessment

Demographic data, including height, weight, foot length, and current medication intake were assessed. Cognitive tests included the MMSE (Folstein et al., 1975) and the Montreal Cognitive Assessment (MoCA) (Gill et al., 2008). The Mini Balance Evaluation Systems Test (Mini-BESTest) (Horak et al., 2009) served as a general measure of balance. In patients with PD, disease severity was assessed by the Movement Disorder Society Unified Parkinson’s Disease Rating Scale (MDS-UPDRS) part III (motor examination) and the Modified Hoehn and Yahr staging (Goetz et al., 2008). Clinical phenotypes were based on calculations using MDS-UPDRS items 2.10, 3.15–3.18 for tremor dominant (TD) versus items 2.12, 2.13, 2.10–3.12 for postural instability/gait difficulty (PIGD) parkinsonism (Stebbins et al., 2013). Tremor of the lower limbs was also examined as this may have an influence on proprioceptive capacity (MDS-UPDRS item 3.17c + d) (Stebbins et al., 2013). The presence and severity of FOG was evaluated by means of the New Freezing of Gait-Questionnaire (NFOG-Q) (Nieuwboer et al., 2009). All tests were performed while patients were optimally medicated.

#### Position sense

Joint position sense was tested with a lower limb contralateral matching task in order to identify possible proprioceptive differences between both groups of fallers (Lord et al., 2003). One lower limb was brought to a random position by the investigator and participants were instructed to match this position with the other limb. During this task, participants were sitting on a chair with their eyes closed (EC). A VICON motion capture system with six cameras and sampling frequency of 100 Hz recorded all movements in a 3D space via reflective markers attached to anatomical landmarks of the participant’s body. Data were analyzed with VICON workstation software, Visual 3D software (C-motion), and Microsoft Excel 2010. Differences in position matching were measured for a range of three knee angles for both limbs and expressed as an average error score in degrees.

#### Assessment of postural stability

Postural stability was assessed by means of a Computed Assisted Rehabilitation Environment (CAREN) platform with integrated dual force plates (AMTI, Watertown, USA) and D-flow software (Motek Medical BV, Amsterdam, The Netherlands). For all posturographic tests, participants were tested while standing barefoot on the force plates, with each foot on a separate plate. To maintain a consistent stance position over trials, foot outlines were marked in such a way that all trials were performed at the same stance width (15 cm between the feet at an angle of 10°). This stance position was experienced as most comfortable based on pilot tests. Participants were asked to stand upright with their arms crossed over the chest. In order to prevent falls, all participants were secured by a safety harness connected to the ceiling.

During the first posturographic test, the perceived LoS were determined by instructing participants to maximally lean in four directions, i.e., forwards, backwards, to the left, and to the right. Participants were asked to lean as far as possible, without lifting their heels, toes or feet, and without flexing their hips.

Secondly, the contribution of each sensory system to the participants’ postural control was assessed under six conditions comprising a combination of visual, vestibular, and proprioceptive manipulations. Participants were asked to maintain quiet standing during three different stance conditions, each performed on: (i) a stable surface with eyes open (EO) and with EC, (ii) on an unstable surface (NeuroCom foam pad, dimensions 45 cm × 45 cm, thickness 13 cm, medium density) with EO and EC, and (iii) during ankle tendon vibration with EO and with EC. Tendon vibration was given on the Achilles tendon of each foot at a frequency of 35 Hz (custom-made vibrators).

Standing on the stable and unstable surface lasted 40 s of which 20 s with EO and 20 s with EC after a visual signal was given. The condition with Achilles tendon vibration had a total duration of 40 s for both EO and EC, of which tendon vibration was turned off during the first 20 s and turned on during the next 20 s. All conditions were executed in random order and each single test was repeated three times. A short period of rest was provided between all tests to avoid muscle fatigue.

### Posturographic outcomes

The main outcomes, CoP displacements, referred to as postural sway, and perceived LoS, were determined in anterior–posterior (AP) and ML directions separately. AP and ML CoP displacement was derived from the difference between vertical forces in AP and ML directions. Force signals were sampled at 3000 Hz. LoS were quantified as the maximal amount of postural sway over the base-of-support (i.e., the feet when standing) that a person can tolerate without falling or having to take a step (Frenklach et al., 2009). LoS in AP and ML direction were derived from the difference between maximal CoP displacement during forward versus backward and left versus right maximal leaning, respectively. For all variables, the average of the three trials was taken, with the exception of some missing values as a result of severe instability or falls. The maximal AP and ML CoP displacements were expressed as absolute values (indicated as absolute sway). Sway values in the AP direction were normalized as a percentage of foot length. In addition, sway outcomes were also expressed as a percentage of their respective LoS (indicated as relative sway).

### Statistical analysis

Data were analyzed using STATISTICA (Statistical analysis Software, version 10). Clinical parameters were compared between groups (fallers with and without PD) with a Mann–Whitney *U* test as data where not normally distributed. The sway and relative sway in AP and ML direction were analyzed by means of a two groups × three stance conditions × two visual conditions repeated measures ANOVA’s. *Post hoc* comparisons of means were conducted by means of Tukey’s honestly significant difference (HSD) test. LoS in AP and ML direction and joint position sense (JPS) were compared between groups by means of *t*-tests for independent samples. *P*-values lower than 0.05 were considered significant. *t*-Tests were reported using Cohen’s *d* with a small effect reflected as 0.2, a medium effect as 0.5, and a large effect as 0.8 (Cohen, 1988). Effect sizes for all ANOVA’s were reported using partial eta squared $\left(\eta_{p}^{2}\right)$. According to Cohen (1988) small, medium, and large effects were defined as 0.0099, 0.0588, and 0.1379, respectively.

## Results

### Participants demographics

Participants’ socio-demographic characteristics are presented in Table 1. No significant differences were found regarding age, fall frequency and scores on MMSE, MoCA, and Mini-BESTest (*p* > 0.05). PD patients had a median disease duration of 13 [Interquartile range (IQR) 25–75% = 6–18] years, UPDRS III score of 40 (28–48) and were in Hoehn and Yahr stage 2 or 3. Patients predominantly met the criteria for the PIGD phenotype (*n* = 9; TD, *n* = 3; indeterminate, *n* = 1). Two patients suffered from tremor in the lower extremities [score 1.5 (1–2)]. Nine patients of the PD group suffered from FOG [NFOG-Q = 19 (10–21)].

**Table 1:** **Subject characteristics; median and interquartile range (25–75%)**.

Parameter	Elderly fallers	PD fallers	*p*-value
Age (years)	74 (71–77)	72 (71–75)	0.489
Disease duration (years)	–	13 (6–18)	–
Fall frequency	2 (2–3)	3 (2–5)	0.293
MMSE (0–30)	29 (28–29)	28 (28–29)	0.317
MoCA (0–30)	27 (23–28)	24 (23–25)	0.209
Mini-BESTest (0–32)	26 (20–29)	21 (18–23)	0.073
UPDRS III (0–108)	–	40 (28–48)	–
UPDRS tot (0–199)	–	77 (60–82)	–
NFOG-Q tot (0–28)	–	19 (10–21)	–

*MMSE, Mini-Mental State Examination; MoCA, Montreal Cognitive Assessment; Mini-BESTest, Mini Balance Evaluation System Test; UPDRS, Unified Parkinson’s Disease Rating Scale; NFOG-Q tot, total score on the New Freezing Of Gait Questionnaire*.

Three PD patients were not able to complete all force plate assessments yielding missing values in specific conditions as a result of severe instability and near-falls. One patient was not able to perform the maximal leaning task in the posterior direction and to maintain standing on the foam, while two patients were not able to stand on the foam with EC of which one also could not complete the condition with Achilles tendon vibration.

### Position sense

For the JPS task no significant differences were found in error scores between elderly and PD fallers for both right (EF: 6.95° ± 7.30; PD: 6.20° ± 6.25) (*p* = 0.780, *d* = 0.111) and left (EF: 6.66° ± 7.09; PD: 5.91° ± 6.16) (*p* = 0.775, *d* = 0.114) lower limb matching. As well, no differences in JPS were found between PD patients’ most (5.65° ± 5.26) and least (6.46° ± 7.00) affected side (*p* = 0.743, *d* = 0.130).

### Limits of stability

Parkinson’s disease and EF had similar perceived LoS. In the AP direction, expressed as a percentage of foot length, the average LoS in PD patients was 61.30% (±13.83) and in EF 63.11% (±7.78) (*p* = 0.688, *d* = 0.161). In the ML direction, expressed in centimeter, PD patients had perceived LoS of 26.40 (±6.03) cm and EF of 27.51 (± 3.39) cm (*p* = 0.57, *d* = 0.228).

### Postural sway

#### Medio-lateral direction

**Figure 1:**
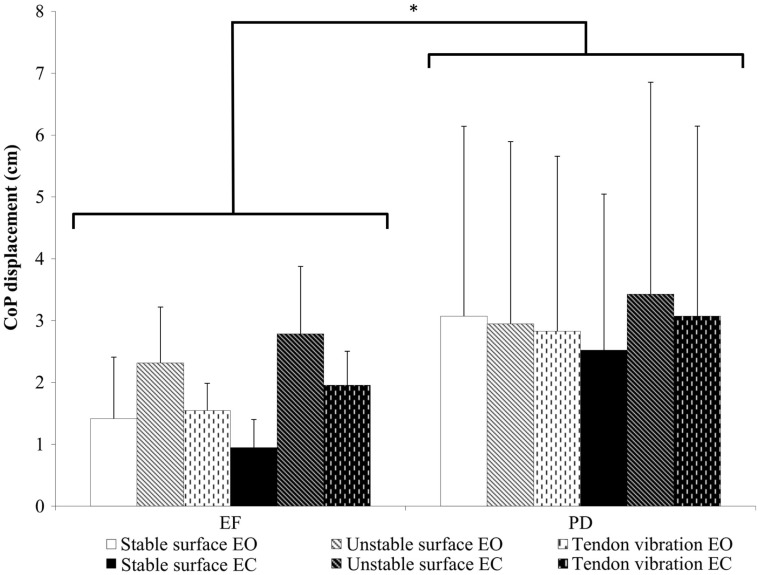
**CoP displacement (centimeter) in ML direction ± SD**. A significant main effect was found, indicating higher postural sway in the PD group across all conditions (*p* = 0.043). Also a significant interaction effect was found for condition × vision (*p* < 0.0001) (not in figure, see Supplementary Material). *p ≤ 0.05.

In the ML direction, the group × stance × vision interaction was not significant (*p* = 0.968, $\eta_{p}^{2}=0.002$). A significant main effect was observed for group (*p* = 0.043, $\eta_{p}^{2}=0.182$), showing larger postural sway in PD fallers than in EF across all conditions (Figure 1). As well, a significant interaction effect of stance by vision was found (*p* < 0.0001, $\eta_{p}^{2}=0.375$). *Post hoc* analysis showed that both groups performed worse in the conditions without vision when standing on the unstable surface and when standing with Achilles tendon vibration. Detailed results of *post hoc* tests are provided in Tables S1 and S2 in Supplementary Material.

#### Anterior–posterior direction

**Figure 2:**
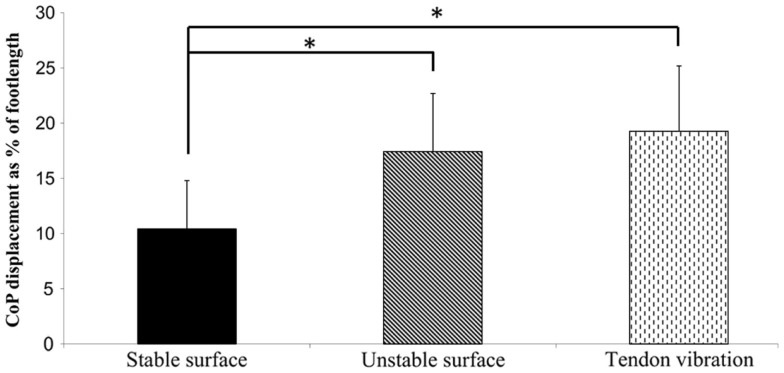
**Average absolute sway of elderly and PD fallers in AP direction ± SD**. A significant effect of condition was found (*p* = 0.000119), indicating that postural sway on the unstable surface and with Achilles tendon vibration was higher compared to standing on the stable surface. *p ≤ 0.001.

In the AP direction no significant group × stance × vision interaction was found (*p* = 0.239, $\eta_{p}^{2}=0.066$). A significant main effect was found for stance condition (Figure 2). *Post hoc* tests showed an increase in postural sway when standing on the unstable surface and in the presence of tendon vibration in comparison to standing on a stable surface (*p* < 0.001). As well, a main effect of vision was present (*p* < 0.001), with all participants having greater CoP displacement in the conditions with EC compared to EO. In contrast to the ML direction, in AP the stance condition × vision interaction was not significantly different (*p* = 0.062, $\eta_{p}^{2}=0.124$). Detailed results of *post hoc* tests are provided in Tables S1 and S3 in Supplementary Material.

### Relative postural sway

**Figure 3:**
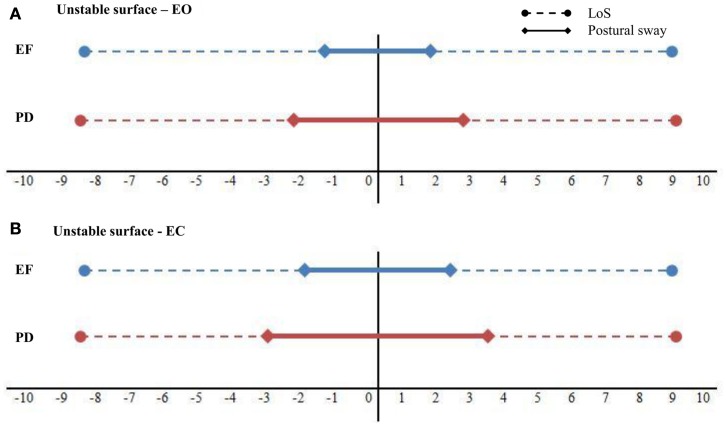
**Individual plots of AP postural sway in relation to the perceived limits of stability in a representative subject form each group during the unstable standing condition with EO (A) and EC (B)**. **(A)** For the EF 17.93% of the LoS are taken up by sway, compared to 28.42% in the PD faller. **(B)** During standing with EC 24.72% of the LoS is taken up by postural sway in the EF, 36.89% in the PD faller.

**Figure 4:**
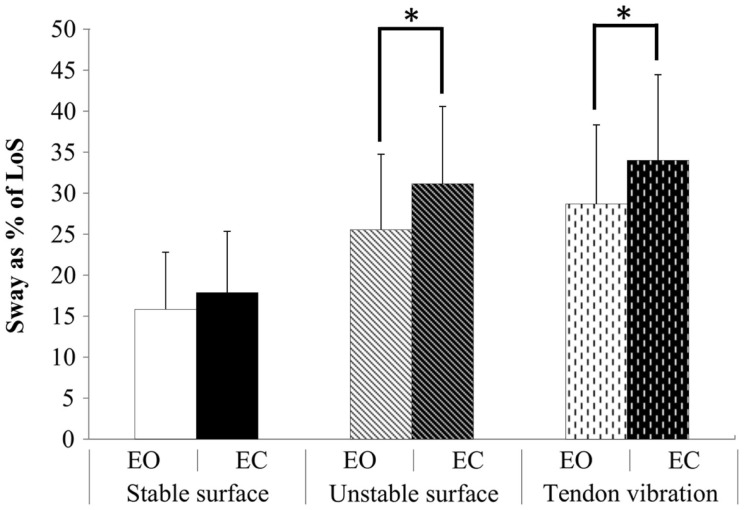
**Average relative sway of elderly and PD fallers in AP direction ± SD**. A significant interaction effect for condition × vision was found (*p* = 0.043), showing increased postural sway upon proprioceptive disturbances, which increased further in the absence of vision. *p ≤ 0.05.

Figures 3A,B show the individual plots of postural sway in relation to the perceived LoS in a representative subject from each group during EO and EC. This figure illustrates how the percentage of the respective LoS is taken up by postural sway during standing on the unstable surface. Here, the relative postural sway in AP direction is illustrated. Although these results were not significant, analysis did show a strong trend that PD patients swayed more closely to their stability limits compared to EF (*p* = 0.05, $\eta_{p}^{2}=0.170$) in all ML conditions. Comparing group data of the absolute amount of postural sway as percentages of the perceived LoS, comparable results were found for the ML and AP direction, showing no significant group × stance × vision interaction (*p* = 0.897, $\eta_{p}^{2}=0.005$; *p* = 0.270, $\eta_{p}^{2}=0.060$, respectively). We also found a significant interaction effect for stance condition × vision in AP direction (*p* = 0.043, $\eta_{p}^{2}=0.139$) (Figure 4), indicating that in both groups the amount of postural sway within the perceived LoS was higher when standing with tendon vibration than while standing on a stable surface when visual feedback was reduced (EC). See Tables S1–S3 in Supplementary Material for detailed results of *post hoc* tests.

## Discussion

This study investigated the impact of proprioceptive manipulations on postural control in patients with PD and healthy people with a history of falls. Comparing a group of PD patients with EF, instead of elderly non-fallers allowed identifying both general aspects of postural imbalance and additional Parkinson-specific components. Both groups responded with higher postural sway in conditions without vision and in conditions when the proprioceptive input was manipulated. This finding indicates that EF and PD patients have a similar weighting of ankle proprioceptive information for postural control. Overall, the findings did not confirm our primary hypothesis that greater basal ganglia dysfunction and greater sensorimotor integration deficits would lead to more sway in PD during proprioceptive manipulations. However, PD fallers did respond with greater postural sway in the ML direction across all conditions irrespective of manipulations. These results are even more striking as PD patients and healthy controls had a similar history of falling, did not differentiate on clinical balance tests, had similar cognitive profiles and were of a similar age. As such, the results point toward a Parkinson-specific problem in maintaining ML stability, which most likely has a motor origin although a contribution of the proprioceptive system cannot be completely ruled out.

In contrast to the present study, most previous studies compared fallers and non-fallers in order to identify postural mechanisms related to falls (Melzer et al., 2004; Ghulyan and Paolino, 2005; Mujdeci et al., 2012; Muir et al., 2013). Muir et al. (2013) reported a larger CoP displacement in fallers compared to healthy elderly. Dynamic posturography revealed more pronounced lateral balance deficits in older adults with a history of falls compared to those without (Ghulyan and Paolino, 2005). EF also showed impaired balance control during narrow stance (Melzer et al., 2004), in a near-tandem stability test (Lord et al., 1999) and in both spontaneous – and induced-sway tests (Maki et al., 1994) compared to non-fallers, as evidenced by an increased ML sway in the group with a history of falls. According to previous comparisons between EF and non-fallers, it is suggested that increased ML sway could be an important predictor for future risk of falling.

Other studies, on the other hand, have investigated differences in postural control between PD patients and healthy controls without a fall history. Intriguingly, also in these studies, ML sway measures were found to be more sensitive to assess balance control in PD patients compared to healthy controls (Mitchell et al., 1995; Mancini et al., 2012).The present study extended these findings by showing that PD fallers had even more postural sway in ML direction compared to EF with a fall history, suggesting that higher ML sway measures may particularly contribute to balance disorders in PD.

The high fall risk in both EF and PD patients is also associated with muscle weakness, contributing to reduced postural stability (Latt et al., 2009). Moreover, it is suggested that PD is associated with impaired ML gain control and that this may contribute to balance impairment (Carpenter et al., 2004). This directionally specific postural instability may be due to a muscle stiffening response (Carpenter et al., 2004; Horak et al., 2005; Chastan et al., 2008), which hampers switching from the ankle inverted pendulum strategy to a hip strategy during postural perturbations.

Interestingly, in the present study, no differences were found regarding the perceived LoS between the two groups. In contrast, Mancini et al. (2008) found reduced LoS in PD patients in comparison to controls during voluntary maximal forward and backward leaning. The result of our study therefore may reflect the fact that both groups consisted of fallers. An interesting finding was that we found a strong trend indicating that PD fallers swayed more closely to their perceived LoS in ML direction. This mismatch between perceived LoS and actual sway potentially explains the high fall risk in PD. However, fall frequency was equal in both groups in this study. These results support findings by Menant et al. (2011) comparing healthy elderly and PD patients “on” and “off” levodopa. These authors not only found reduced stability margins in the PD groups, but also a significantly higher sway as a percentage of their LoS, regardless of medication state in patients with PD. Horak et al. (2005) found that PD patients had smaller stability margins compared to age-matched controls, by investigating their postural responses to surface translations, a task which heavily depended on proprioception. In the present study, PD fallers responded with greater postural sway in the ML direction compared to EF across all conditions, irrespective of sensory manipulations.

In both groups of fallers we found a significant effect of visual and proprioceptive disturbances upon balance performance in ML and AP direction. Postural sway typically increased in the absence of visual input or disturbed proprioceptive feedback, which is in line with several studies pointing to an increased reliance on external visual or on proprioceptive information for the control of posture and locomotion in elderly subjects (Lord et al., 1994, 1999; Liaw et al., 2009; Ickenstein et al., 2012) and even more so in PD (Azulay et al., 1999; Colnat-Coulbois et al., 2011). Results of Lord et al. (1994, 1999) showed increased lateral sway as well as poorer visual acuity, quadriceps strength, and proprioception in elderly with a history of falls (Lord et al., 1994, 1999). The comparison group in our study consisted of healthy older fallers with similar proprioceptive ability as the PD group, which may explain the similar response to visual or proprioceptive manipulation.

Previous studies showed disagreement about the directions in which postural sway increased. In the current study, postural sway increased in ML and AP directions in both groups in conditions with sensory disturbances, but the extent of the abnormality depended on the sensory condition. ML sway increased to a higher extent when standing on an unstable surface compared to the tendon vibration condition, whereas AP sway increased more when tendon vibration was applied compared to standing on the unstable surface. This apparent contradiction may be explained by the manner in which both sensory manipulation techniques alter proprioception. When Achilles tendon vibration is applied, muscle spindles respond as if the vibrated muscle is stretched, causing an illusory forward body movement, resulting in a compensatory backward movement (Vaugoyeau et al., 2011). The foam surface, on the other hand, decreases a person’s ability to sense pressure distribution and reduces the effectiveness of ankle torque required for postural stabilization (Patel et al., 2008). An unequal distribution of pressure between the feet may cause postural instability and to overcome this, the CoP needs to be shifted between the left and right foot resulting in an increased ML sway. As foam properties (i.e., thickness and density) also influence the amount of postural sway and task difficulty (Maitre et al., 2014), the foam used was possibly equally challenging for both groups to provoke significant differences between groups.

In accordance with earlier research, the current findings suggest an increased dependence on visual input and proprioceptive information of the ankles in both elderly and PD fallers. Sensorimotor integration is facilitated by the basal ganglia, which receive visual and proprioceptive information. Brain imaging studies showed that healthy aging is associated with reduced basal ganglia activity during the performance of a proprioceptive task (Goble et al., 2011; Suetterlin and Sayer, 2013). In patients with PD, dopamine depletion may negatively affect this information processing even more (Konczak et al., 2009). However, our findings do not support the idea that stability differences between elderly and PD fallers are underpinned by proprioceptive difficulties, but may instead reflect disease-specific motor deficits.

Basal ganglia dysfunction may not be the only explanation underlying postural instability in PD. In contrast to the cardinal motor symptoms of PD (bradykinesia, akinesia, rigidity, and tremor), postural instability hardly improves and even worsens as a result of dopaminergic medication (Bronte-Stewart et al., 2002; Shivitz et al., 2006; Chastan et al., 2008; Frenklach et al., 2009; Yarnall et al., 2011). Recently, attention is drawn to the role of cholinergic structures in the etiology of gait and balance problems (Bohnen et al., 2009; Gilman et al., 2010; Rochester et al., 2012). Bohnen et al. (2009) compared PD fallers, PD non-fallers, and controls in a PET study and found significantly lower cortical and thalamic acetylcholine uptake in PD fallers. The peduncolopontine nucleus supplies the majority of the cholinergic input to the thalamus (Yarnall et al., 2011) and its neural degeneration is perceived as a major factor leading to impaired postural control in PD (Bohnen et al., 2009).

A recent study by Tuunainen et al. (2014) found that a higher fall risk in the elderly population was also correlated with fear of falling, which is a common problem in both EF and PD patients (Bryant et al., 2013). There is also increasing attention for how physical activity levels may affect postural instability, as several studies reported positive effects of physical training on balance, falls and proprioceptive acuity [for review see Suetterlin and Sayer (2013)]. Recent research of Maitre et al. (2014) showed that Achilles tendon vibration disturbed postural control in older subjects, but that regular physical activities could enhance their ability to withstand postural disturbance. In the current study, we did not control for activity levels of the participants, which may be a potential limitation. We also used tendon vibration of only one frequency and one temporal interval at solely one muscle group. A more systematic use of different vibration frequencies and temporal intervals may have led to stronger differences. For future studies it might also be interesting to analyze changes in postural sway over several time-series to assess the ability to timely adapt to sensory manipulation, as previously conducted by Doumas and Krampe (2010). As well, future studies should investigate larger groups of patients as our current results were weakened by a rather small sample size and high variability, leading to limited effect sizes. As well, three PD patients were not able to complete all assessments due to severe instability or near-falls and their data could not be included in analysis. Furthermore, as FOG is a powerful determinant of falls (Bloem et al., 2004; Latt et al., 2009) and has been associated with greater proprioceptive deficits in a previous study (Tan et al., 2011), future research may benefit from investigating the role of proprioception in falls and postural stability in freezers and non-freezers separately. A major limitation of the present study is that we did not include a group of healthy elderly without a history of falls. Postural sway increased in both group of fallers during conditions when proprioception was disturbed, suggesting a similar use of ankle proprioception input in both groups. However, from these results it is unclear whether fallers make more use of an ankle-steered postural control strategy compared to non-fallers and consequently are at higher risk for falls. Therefore, we need to interpret our results with caution and require additional research on the reliance of ankle proprioception in the future.

The current study aimed to investigate the effect of proprioceptive manipulations on postural control in patients with PD and EF. As Achilles tendon vibration led to similar response in all fallers, our findings suggest a similar reliance on ankle proprioceptive information in fallers irrespective of PD. PD fallers responded with greater postural sway in ML direction upon proprioceptive manipulations disturbing postural control, as a result of which these values approached the perceived LoS more closely than in EF. We conclude that despite a similar fall history, PD patients show more ML instability than EF irrespective of sensory manipulations, but show a similar reliance on ankle proprioceptive information for postural control. Focusing on motor rather than on sensory aspects might therefore be more beneficial in rehabilitation programs and fall prevention training for patients with PD.

## Conflict of Interest Statement

The authors declare that the research was conducted in the absence of any commercial or financial relationships that could be construed as a potential conflict of interest.

## Supplementary Material

The Supplementary Material for this article can be found online at http://www.frontiersin.org/Journal/10.3389/fnhum.2014.00939/abstract
