# Supplementary material for: The Contribution of Proprioceptive Information to Postural Control in Elderly and Patients with Parkinson’s Disease with a History of Falls
Source: Front Hum Neurosci. 2014 Nov 24;8:939. doi: 10.3389/fnhum.2014.00939 (PMC4241823; doi:10.3389/fnhum.2014.00939)
Supplement: Supplementary file 1 [file Data_Sheet_1.PDF]

Supplementary Material

The contribution of proprioceptive information to postural control in elderly and patients with Parkinson's disease with a history of falls

Esther M.J. Bekkers[1]*, Kim Dockx[1], Elke Heremans[1], Sarah Vercruysse[1], Sabine M.P. Verschueren[2], Anat Mirelman[3], Alice Nieuwboer[1]

[1]Neuromotor Rehabilitation Research Group, Department of Rehabilitation Sciences, KU Leuven, Belgium
[2]Musculoskeletal Rehabilitation Research Group, Department of Rehabilitation Sciences, KU Leuven, Belgium
[3]Movement Disorders Unit, Department of Neurology, Tel-Aviv Sourasky Medical Center, Israel

*** Correspondence:** Esther M.J. Bekkers, Neuromotor Rehabilitation Research Group, Department of Rehabilitation Sciences, KU Leuven, Tervuursevest 101 box 1501, Heverlee, B-3001, Belgium.
esther.bekkers@faber.kuleuven.be

1. Supplementary Figures and Tables

1.1. Supplementary Tables

Supplementary Table 1. Absolute and relative postural sway in ML and AP direction for all participants, mean ± SD

	ML Absolute sway (cm)	ML Relative sway (%)	AP Absolute sway (%)	AP Relative sway (%)
Stable surface EO	2.24 ± 2.98	8.51 ± 10.83	9.90 ± 4.50	15.75 ± 7.21
Stable surface EC	1.73 ± 2.38	6.68 ± 8.24	10.95 ± 5.04	17.73 ± 8.17
Unstable surface EO	2.62 ± 1.01	9.83 ± 4.10	15.34 ± 4.35	25.50 ± 9.19
Unstable surface EC	3.06 ± 1.06	11.09 ± 3.81	19.53 ± 6.39	31.19 ± 9.92
Tendon vibration EO	2.16 ± 1.46	8.44 ± 5.67	20.82 ± 5.86	28.51 ± 9.61
Tendon vibration EC	2.49 ± 1.20	9.65 ± 4.64	62.24 ± 10.90	33.98 ± 10.17

Supplementary Table 2. Detailed results of post-hoc tests in ML direction

		**Absolute sway** ***p*-value**	**Relative sway** ***p*-value**
Stable surface EO	Stable surface EC	0.013385 *	0.016269 *
	Unstable surface EO	0.067677	0.018395 *
	Unstable surface EC	0.000156 ***	0.000144 ***
	Tendon vibration EO	0.951684	0.991830
	Tendon vibration EC	0.494846	0.209447
Stable surface EC	Unstable surface EO	0.000142 ***	0.000142 ***
	Unstable surface EC	0.000141 ***	0.000141 ***
	Tendon vibration EO	0.112165	0.069405
	Tendon vibration EC	0.000193 ***	0.000151 ***
Unstable surface EO	Unstable surface EC	0.083449	0.084838
	Tendon vibration EO	0.007189 *	0.003705 *
	Tendon vibration EC	0.883880	0.893378
Unstable surface EC	Tendon vibration EO	0.000142 ***	0.000142 ***
	Tendon vibration EC	0.005042 *	0.00519 *
Tendon vibration EO	Tendon vibration EC	0.109910	0.061336

* $p \leq 0.05$; ** $p \leq 0.01$; *** $p \leq 0.001$

Supplementary Table 3. Detailed results of post-hoc tests in AP direction

		**Absolute sway, normalized** *p***-value**	**Relative sway** *p***-value**
Stable surface EO	Stable surface EC	0.824261	0.756060
	Unstable surface EO	0.000143 ***	0.000142 ***
	Unstable surface EC	0.000141 ***	0.000141 ***
	Tendon vibration EO	0.000141 ***	0.000141 ***
	Tendon vibration EC	0.000141 ***	0.000141 ***
Stable surface EC	Unstable surface EO	0.000263 ***	0.000203 ***
	Unstable surface EC	0.000141 ***	0.000141 ***
	Tendon vibration EO	0.000141 ***	0.000141 ***
	Tendon vibration EC	0.000141 ***	0.000141 ***
Unstable surface EO	Unstable surface EC	0.000490 ***	0.000263 ***
	Tendon vibration EO	0.127137	0.087044
	Tendon vibration EC	0.000142 ***	0.000141 ***
Unstable surface EC	Tendon vibration EO	0.282528	0.222602
	Tendon vibration EC	0.412667	0.266027
Tendon vibration EO	Tendon vibration EC	0.002836 *	0.000898 ***

* $p \leq 0.05$; ** $p \leq 0.01$; *** $p \leq 0.001$
